# DeepKOALA: a scalable deep learning framework for KEGG Orthology assignment

**DOI:** 10.1093/bib/bbag445

**Published:** 2026-08-19

**Authors:** Zhaoxi Yu, Lingjie Meng, Canh Hao Nguyen, Hiroshi Mamitsuka, Minoru Kanehisa, Hiroyuki Ogata

**Affiliations:** Bioinformatics Center, Institute for Chemical Research, Kyoto University, Gokasho, Uji, 611-0011, Japan; Bioinformatics Center, Institute for Chemical Research, Kyoto University, Gokasho, Uji, 611-0011, Japan; Bioinformatics Center, Institute for Chemical Research, Kyoto University, Gokasho, Uji, 611-0011, Japan; Bioinformatics Center, Institute for Chemical Research, Kyoto University, Gokasho, Uji, 611-0011, Japan; Bioinformatics Center, Institute for Chemical Research, Kyoto University, Gokasho, Uji, 611-0011, Japan; Bioinformatics Center, Institute for Chemical Research, Kyoto University, Gokasho, Uji, 611-0011, Japan

**Keywords:** KEGG Orthology, deep learning, metagenomics, protein function annotation

## Abstract

The KEGG Orthology (KO) system links DNA and protein sequences to biological functions and pathways, providing a curated, fundamental, and consistent annotation framework across all domains of life. While accurate, traditional sequence alignment-based annotation methods are computationally expensive, which severely limits their application in large-scale datasets. To address this challenge, we introduce Deep KEGG Orthology and Links Annotation (DeepKOALA), a deep learning approach based on Gated Recurrent Units (GRU), which frames KO annotation as an open-set recognition task. This design reduces false positives arising from out-of-scope sequences and, together with a lightweight GRU backbone, enables high-throughput annotation. The GRU-based model was benchmarked against four other deep learning architectures and showed the best balance between speed and accuracy. We then trained a GRU-based model, DeepKOALA, and performed a cross-species evaluation against existing KO annotation tools. In this comparison, DeepKOALA achieved a F1 of 83.37%, which is comparable to existing alignment-based tools. Meanwhile, the speed of DeepKOALA was 36.5-fold faster than Blast KEGG Orthology and Links Annotation (BlastKOALA). We also provide a specialized fragment model for handling incomplete sequences and an optional multi-domain mode. Together, these features make DeepKOALA a scalable and efficient option for high-throughput function annotation.

## Introduction

The Kyoto Encyclopedia of Genes and Genomes (KEGG) and KEGG Orthology (KO) systems provide foundational resources for the systematic understanding of gene functions [1]. Built through manual curation, the KO system links genes to diverse biological data organized in an experiment-grounded comprehensive framework. Current KO annotation relies on two sequence-alignment-based approaches. The first detects homologous sequences via pairwise sequence alignment—e.g. Basic Local Alignment Search Tool (BLAST)-based workflows, such as Blast KEGG Orthology and Links Annotation (BlastKOALA) and Ghost KEGG Orthology and Links Annotation (GhostKOALA) [2]—whose runtime scales with the reference database size. The second approach scans sequences against KO-specific profile hidden Markov models (HMMs)—e.g. KofamKOALA/KofamScan, the web-based and local command-line implementations of a KO family profile HMM-based KEGG Orthology And Links Annotation method [3]—whose runtime scales with the total number of KO profiles, thus reducing the computational cost. However, these alignment-based methods face a serious computational bottleneck for large-scale analyses. This challenge is particularly acute in metagenomics, which produces petabyte-scale datasets comprising vast sequence spaces of proteins inside and those outside current annotation coverage. There is therefore an urgent need for an annotation approach that is both efficient and accurate at scale.

Recent advances in computational biology have introduced a range of deep-learning-based architectures for protein sequence modeling and protein family classification, including Convolutional Neural Networks [4], Recurrent Neural Networks (RNN) [5], Gated Recurrent Units (GRU) [6], Long Short-Term Memory (LSTM) [7], state-space models (e.g. Mamba [8]), and large protein language models (e.g. Evolutionary Scale Modeling 2 (ESM-2) [9]). Many of these efforts have focused on Gene Ontology (GO) annotation, with representative methods such as DeepGO [10], DeepGOPlus [11], DeepFRI [12], TALE [13], ProLanGO [14], DeepGraphGO [15], NetGO [16], GOLabeler [17], and the zero-shot/neuro-symbolic approaches DeepGOZero [18] and DeepGO-SE [19]. Beyond GO, deep-learning-based models also annotate enzyme EC numbers (e.g. ProteInfer [20] and more recent trustworthy/uncertainty-aware frameworks) and antibiotic resistance genes (e.g. DeepARG [21], HMD-ARG [22]). Although KO remains one of the most widely used systems for pathway-level annotation, existing KO prediction tools rely on sequence alignment and could be improved by deep-learning solutions. Furthermore, most previous alignment-based and machine learning-based approaches formulate prediction as a closed-set problem, assuming that all query sequences belong to known classes, thereby lacking mechanisms for calibrated rejection of out-of-scope sequences [23, 24].

We address this limitation by framing KO annotation as an open-set recognition problem and present Deep KEGG Orthology and Links Annotation (DeepKOALA). To correctly distinguish the sequences that should be assigned to a specific KO from sequences belonging to other KOs and those out of the current KO coverage, we calibrate reliable decision thresholds for each class by jointly leveraging labeled proteins and a large collection of currently unannotated KEGG GENES proteins used as operational out-of-scope examples. We benchmarked RNN, GRU, LSTM, Mamba, and ESM-2 (8 M; checkpoint: esm2_t6_8M_UR50D), and selected GRU as the backbone based on accuracy–efficiency trade-offs and broad central processing unit (CPU)/graphics processing unit (GPU) compatibility. DeepKOALA achieves annotation performance comparable to existing alignment-based tools, while substantially improving throughput. DeepKOALA also includes two practical features for fragmented sequences and multi-domain proteins. We provide a user-friendly web server on GenomeNet (https://www.genome.jp/tools/deepkoala/), downloadable DeepKOALA software on GitHub (https://github.com/zhaoxi120/deepkoala), and regularly updated weights for the models.

## Materials and methods

### Overall workflow

**Figure 1:**
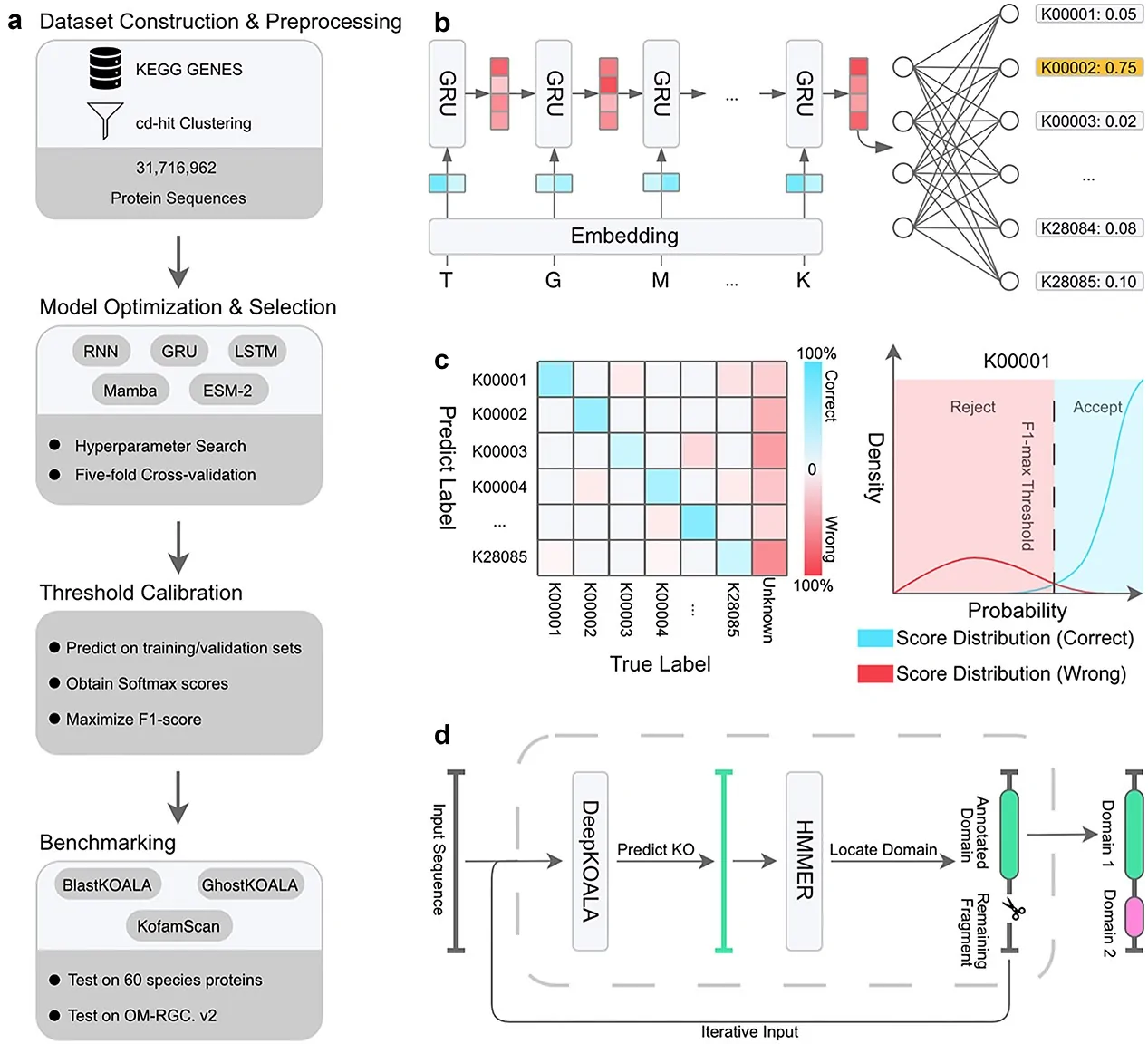
Overview of the DeepKOALA framework; (a) the workflow pipeline comprises four main stages: dataset construction and preprocessing, model optimization and selection via HP optimization, threshold calibration, and final benchmarking against existing annotation tools; (b) the model architecture utilizes an embedding layer and two stacked GRU layers to encode protein sequences, which are then processed by a MLP and Softmax to output KO class probabilities; (c) threshold determination mechanism: the confusion matrix (left) categorizes predictions into correct and wrong groups, while the density plot (right) analyzes their score distributions to identify the optimal threshold maximizing the F1-score; (d) the multi-domain mode employing an iterative hybrid strategy, where DeepKOALA predicts the KO label and HMMER delineates the domain boundary, allowing the annotated domain to be computationally removed and the remaining fragment to be re-analyzed for additional domains.

In the model development phase, we benchmarked several architectures on KEGG sequences and selected a lightweight GRU for its optimal balance of accuracy and efficiency (Fig. 1). We formulated KO assignment as an open-set recognition problem, where the goal is to correctly assign KO annotations while rejecting out-of-scope examples (i.e. sequences without KO annotations in KEGG GENES) using decision thresholds. It should be noted that sequences without KO annotations may be uncovered by the current KO system or simply not yet curated. To determine these thresholds effectively, we calibrated them for each KO class using both labeled and currently unannotated sequences. During the prediction phase, the trained model encodes each input sequence into a fixed-length vector, which is mapped to KO probabilities by a multilayer perceptron (MLP). The top-scoring KO is assigned only if its probability exceeds the calibrated threshold.

### Dataset construction and preprocessing

Our study is framed within the KO system (February 2025), which defines 26 226 functional classes. We utilized the KEGG GENES database (February 2025) with a collection of 55 795 412 protein sequences, of which 52.35% (29 207 030 protein sequences) were annotated with KOs while others were currently unannotated (26 588 382 protein sequences). To mitigate extreme class imbalance, we applied a targeted augmentation strategy for underrepresented KO classes (see Supplementary Method S1 for details).

**Figure 2:**
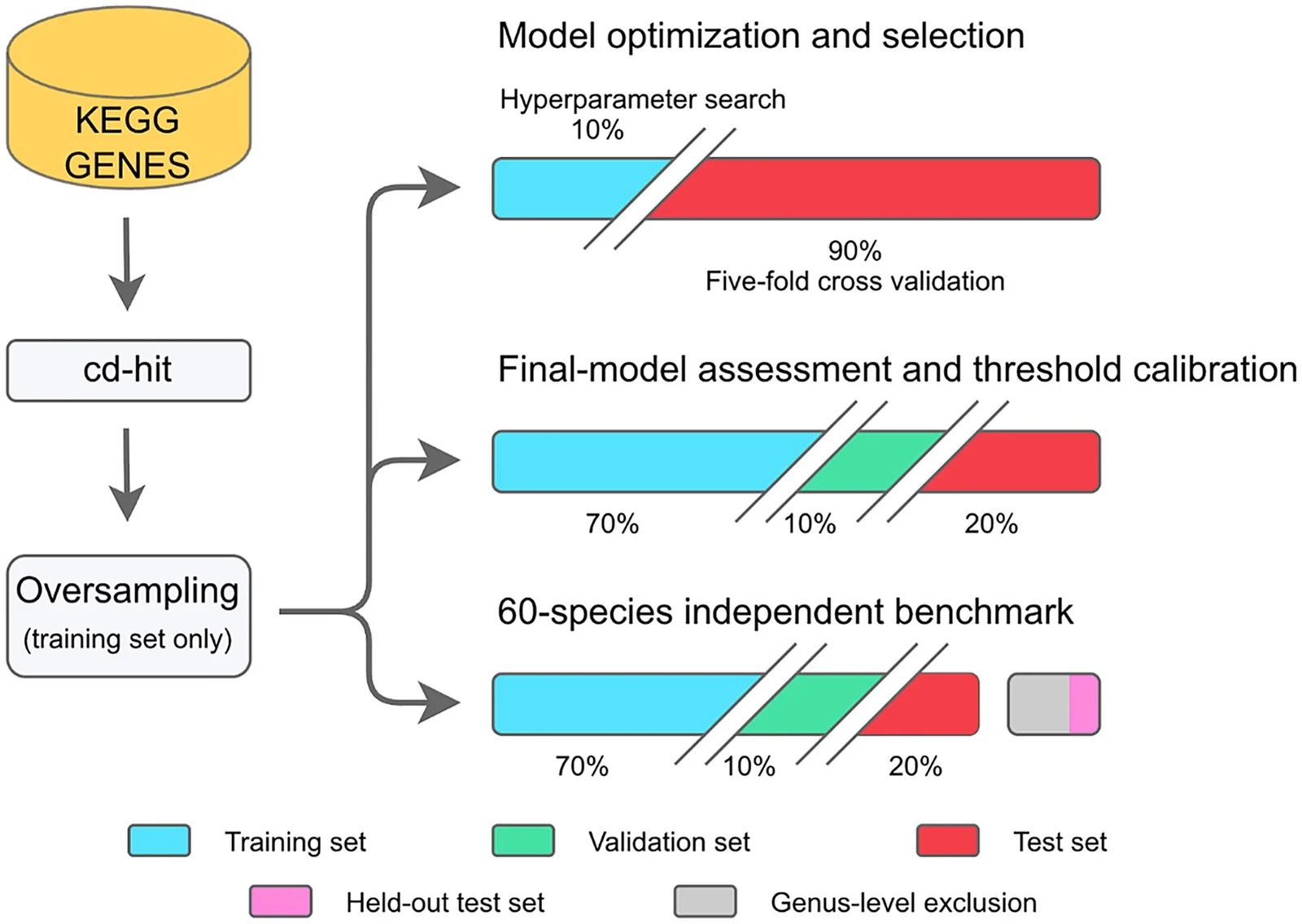
Data splitting strategy for DeepKOALA model development and benchmarking; after CD-HIT de-replication, targeted oversampling was applied only within training sets; the non-redundant dataset was used for model optimization, final-model assessment with threshold calibration, and the independent 60-species benchmark; for the 60-species benchmark, test species were held out, and same-genus sequences were removed from the remaining data and baseline reference databases; colors indicate training, validation, test, held-out independent test, and genus-level exclusion.

To mitigate the bias from sequence redundancy, we used the Cluster Database at High Identity with Tolerance (CD-HIT) [25] tool (with parameter—c 0.9) to cluster the combined dataset and removed redundant sequences with over 90% identity. This resulted in a non-redundant dataset of 31 716 962 sequences (13 682 867 with KO labels and 18 034 095 without), with the sequence-length distribution shown in Supplementary Figure S1. For protein sequences associated with multiple KO labels, we assigned each sequence a single class label corresponding to the unique combination of its KOs (a total of 243 such combination classes). The non-redundant dataset was used in three distinct stages (see Fig. 2), with data splitting and targeted oversampling applied independently within each stage. A standard 7:1:2 ratio for training, validation, and test sets was strictly applied across all partitions except the hyperparameter (HP) search stage. For the full dataset, the minimum class size for oversampling was set to 70 sequences. For each split, this minimum was scaled proportionally to the split size (e.g. 7 sequences for a 10% split and 63 sequences for a 90% split). Targeted oversampling with replacement was applied only within the training splits until the stage-specific minimum was reached, leaving the validation and test sets unchanged (see Supplementary Method S1 for details).

First, for model optimization and selection, the dataset was randomly split at the sequence level into a 10% subset for HP search and a 90% subset for five-fold cross-validation. Because no test set was needed for the HP search, this 10% subset was divided into HP-training and HP-validation sets at a 7:3 ratio. The HP-training set contained only KO-labeled data, whereas the HP-validation set included both KO-labeled data and currently unannotated sequences to support open-set model selection. Currently unannotated sequences were used only as operational out-of-scope examples and did not contribute to gradient updates. The remaining 90% subset was evaluated by five-fold cross-validation. Per-fold training, validation, and test sample counts are provided in Supplementary Table S1.

Second, for final-model assessment and threshold calibration, the complete non-redundant dataset was randomly partitioned. The selected GRU model was trained using this partition. Thresholds were calibrated using a semi-supervised approach incorporating both KO-labeled sequences and currently unannotated sequences from training and validation predictions only, meaning that the test set was strictly excluded from model training, early stopping, HP selection, and threshold calibration. The released production model uses this dataset configuration.

Third, for the 60-species independent benchmark, all protein sequences from 60 randomly selected species were held out as an independent test set. To prevent data leakage, sequences from species in the same genera were removed from the remaining data and from the baseline reference databases. The retained sequences were then partitioned into separate training, validation, and test sets to train and evaluate the benchmark DeepKOALA model.

### Model optimization and selection

Using the data partitions explained above, we systematically compared five candidate models: RNN, GRU, LSTM, Mamba, and ESM-2 (8 M). Model selection involved HP optimization, followed by a five-fold cross-validation.

We first used a dedicated HP tuning set and employed a Bayesian optimization algorithm to find the optimal configuration for each model. This optimization was guided by the Open-set Area Under the Curve (OpenAUC) [26], a threshold-independent metric specifically suited for open-set recognition problems such as the KO annotation task in this study. In this setting, a model must not only classify known KOs but also reject unlabeled examples.

Mathematically, it is defined as:


\begin{align*} OpenAUC=&\frac{1}{\left|{S}_{known}\right|\left|{S}_{unknown}\right|}\sum_{x_i\in{S}_{known}}\sum_{x_j\in{S}_{unknown}}\left[\;1\left[{y}_i=h\left({x}_i\right)\right]\right.\\&\left.\cdotp 1\left[ MSP\left({x}_i\right)> MSP\left({x}_j\right)\right]\;\right] \end{align*}

where ${S}_{known}$ and ${S}_{unknown}$ are the sequence sets with and without KO labels, respectively. $h\left({x}_i\right)$ and ${y}_i$ are the predicted and true labels for ${x}_i$. *MSP* is the maximum Softmax score. In this formula, the model’s performance is evaluated on a pair $\left({x}_i,{x}_j\right)$ of sequences with and without KO labels. When the model both assigns the correct label to the known sequence (i.e. ${y}_i=h\left({x}_i\right)$) and assigns a higher Softmax score to the known sequence compared to the operational out-of-scope example (i.e. $MSP\left({x}_i\right)> MSP\left({x}_j\right)$), we consider this pair as the correct pair. The computed OpenAUC is the probability of a correct pair over all labeled–unlabeled pairs. A higher OpenAUC score thus indicates a proper balance between classification accuracy and the ability to reject operational out-of-scope examples, making it the core metric for our performance evaluation.

To strike a balance between model performance (OpenAUC) and computational efficiency (time), we defined a selection criterion function $f\left( OpenAUC, Time\right)$ to identify the optimal configuration. For each HP combination, we first performed a min–max normalization on the OpenAUC scores and time consumption:


\begin{align*} {OpenAUC}_{norm}=\frac{OpenAUC-{OpenAUC}_{min}}{OpenAUC_{max}-{OpenAUC}_{min}} \end{align*}

\begin{align*} {Time}_{norm}=\frac{Time-{Time}_{min}}{Time_{max}-{Time}_{min}} \end{align*}

We then calculated the cost value $f\left( OpenAUC, Time\right)$ for each configuration, representing its deviation from the ideal scenario (where *OpenAUC=1* and *Time=0*). Reflecting the principle that annotation accuracy is the primary consideration in functional genomics, we assigned a performance weight (${W}_{OpenAUC}$) of 0.8 and a time weight (${W}_{Time}$) of 0.2. The criterion function is defined as:


\begin{align*} f\left( OpenAUC, Time\right)=&{\left({W}_{OpenAUC}\bullet \left(1-{OpenAUC}_{norm}\right)\right)}^2\\&+{\left({W}_{Time}\bullet{Time}_{norm}\right)}^2 \end{align*}

Finally, the configuration that minimized the cost function *f ( OpenAUC, Time)* was selected as the optimal setting for each model. Ideally, the best configuration for each of the four most promising models (RNN, GRU, LSTM, and Mamba) was used for a rigorous five-fold cross-validation on the main evaluation set. The final model architecture was chosen based on the best balance of OpenAUC performance, computational efficiency, and versatility across different hardware environments (CPU/GPU).

### Model framework and algorithm

In the lightweight GRU-based network (Fig. 1b), protein sequences are represented by learned amino-acid embeddings and encoded by two stacked GRU layers. Then the final hidden state is passed to a one-hidden-layer MLP followed by Softmax to obtain KO probabilities.

### Threshold calibration

While the threshold-independent OpenAUC metric is ideal for model comparison, converting the final model into a practical annotation tool requires establishing a specific decision threshold for each KO class. This threshold is applied to the output Softmax probability to define accepted KO assignments and rejected operational out-of-scope predictions.

To determine the optimal threshold for each KO class, we retrained the GRU model, which was selected as the best model among four tested models, by randomly dividing the dataset into three sets: training (70%), validation (10%), and test (20%) sets. Then, we used the trained model to generate predictions and corresponding Softmax scores for all data in the training and validation sets. For each KO class, we focused on the sequences predicted to belong to that class and divided them into two groups: those whose true labels matched the predicted KO class, and those whose true labels differed from it (including sequences annotated with other KO classes or currently unannotated sequences). Based on the Softmax score distributions of these two groups, we determined the final threshold score that maximized the F1-score (the harmonic mean of precision and recall) for the KO class (Fig. 1c and Supplementary Method S2).

### Final performance benchmarking

After selecting the optimal GRU-based architecture, we retrained the benchmark model after the exclusion procedure described below and benchmarked its performance against three KO annotation tools: BlastKOALA, GhostKOALA, and KofamScan. To ensure a rigorous and fair comparison, we generated an independent test gene set of 60 species (*N* = 454 580 sequences), including prokaryotes and eukaryotes. The list of test species and excluded reference species is summarized in Supplementary Tables S3 and S4. All sequences from these species and their genera were strictly removed from the training and validation dataset and the reference databases of all tools prior to evaluation, to prevent data leakage and assess true generalization performance (see Supplementary Method S3). In total, 654 reference species were removed. For KofamScan, the exclusion was applied before multiple alignment and HMM construction. For BlastKOALA and GhostKOALA, the list of 654 species was removed from the KO reference set on internal KEGG deployments.

For the 60-species benchmark, DeepKOALA, BlastKOALA, and KofamScan used 32 CPU cores and 128 GB RAM on a Hewlett Packard Enterprise (HPE) Apollo 2000 with an Intel Xeon Gold 6348 (Ice Lake) CPU; GhostKOALA used 32 CPU cores and 500 GB RAM. Storage was provided by an HPE ClusterStor E1000 system connected to the compute nodes via 200 Gbps InfiniBand. Because this is shared cluster storage rather than storage attached to a single host, individual solid-state drive (SSD)/hard disk drive (HDD) models are not applicable. KofamScan used a February 2025 database, whereas BlastKOALA and GhostKOALA were evaluated on internal KEGG deployments with October 2024 databases. Each 60-species benchmark was repeated three times. The raw repeated measurements and summary statistics are provided in Supplementary Table S10. Local runtimes were measured from Portable Batch System (PBS) resources_used.walltime values, excluding queue time, database downloads, and one-time index builds. For the internal BlastKOALA and GhostKOALA services, client-side wall-clock time from submission to result retrieval was recorded. Software versions and command details are provided in Supplementary Method S3 and Supplementary Table S2.

### Additional features

To enhance the model’s practical applicability, we introduced two auxiliary features:

- **Fragment model (DeepKOALA-fragment):** An independently trained variant model using cropped sequences to improve robustness to incomplete sequences (see Supplementary Method S4).
- **Multi-domain mode:** This mode employs an iterative hybrid strategy, combining the rapid deep learning model with the precise scanning of HMMER [27], a software suite for biosequence analysis using profile hidden Markov models, to achieve comprehensive annotation of multiple functional domains on a single protein (Fig. 1d and see Supplementary Method S4 for the detailed pipeline). For each accepted KO prediction, the corresponding KofamScan HMM profile is used with HMMER to delineate domain boundaries. The top-ranked hmmsearch domain region is excised computationally, and remaining fragments, if these are longer than 50 amino acids, are reanalyzed by DeepKOALA.

## Results

### Model architecture and hyperparameter selection

**Figure 3:**
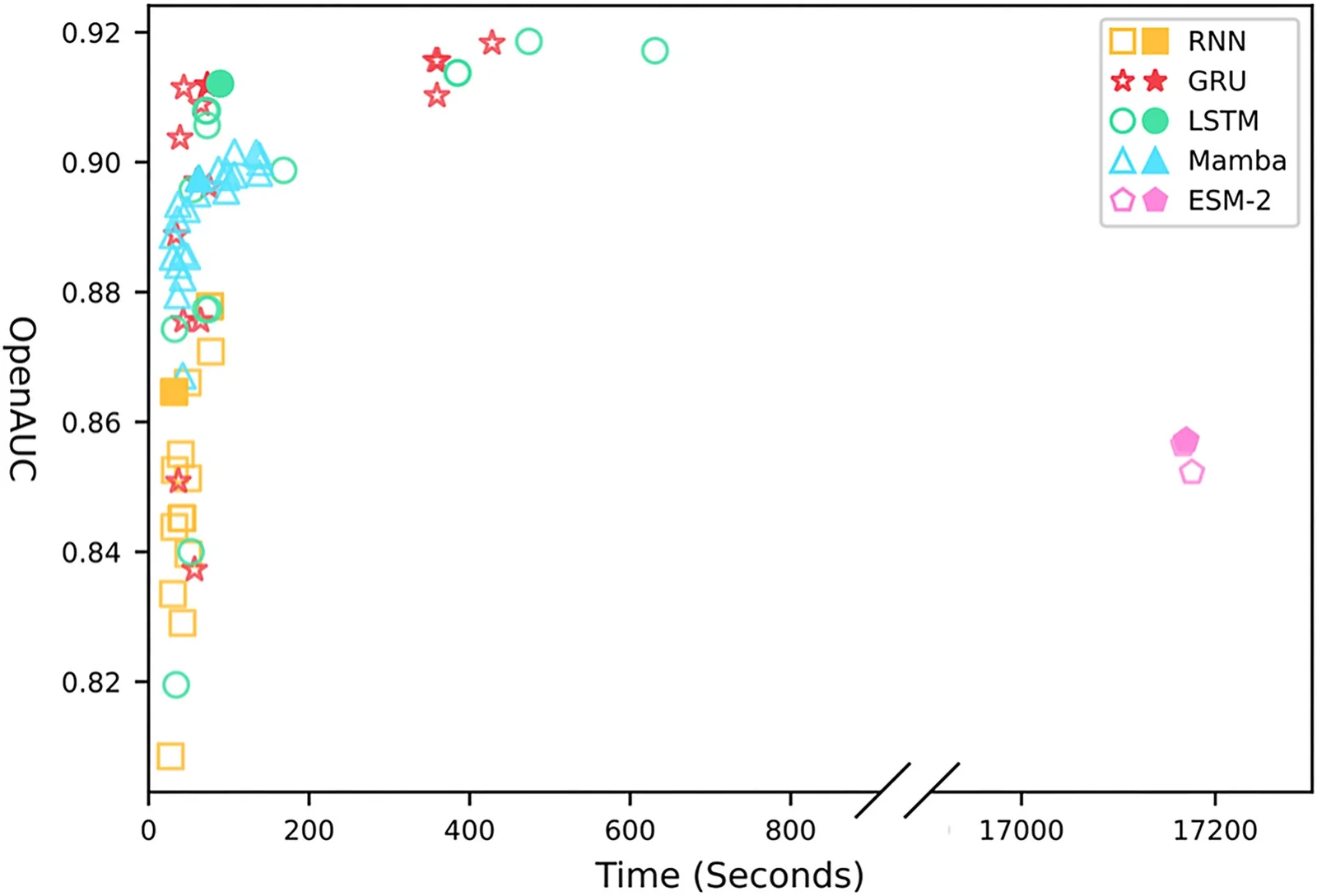
Performance-efficiency distribution from the HP search; the plot shows the performance of the five models under different HP combinations; each data point represents a HP configuration, with its *y*-coordinate as OpenAUC and *x*-coordinate as runtime (seconds); the *x*-axis has a break to show the substantial runtime of ESM-2 (8 M); solid data points indicate the selected optimal configuration for each model based on the cost value; ESM-2 is displayed only as an exploratory reference because its optimization was limited to the final MLP classifier; its values should not be interpreted as directly comparable with the fully optimized lightweight architectures.

To identify the optimal configuration that balances performance and efficiency for four candidate deep learning models (RNN, GRU, LSTM, and Mamba), we conducted a systematic Bayesian optimization search. Key HPs, including the number of stacked layers, hidden units, and the learning rate, were tuned for each model (see Supplementary Method S5 and Supplementary Tables S5–S7). As illustrated in the performance-efficiency trade-offs (Fig. 3), both GRU and LSTM models achieved the highest OpenAUC scores (~0.91) under optimal settings, with the GRU models requiring less execution time. The Mamba model’s performance was close behind with an OpenAUC of 0.90, while the RNN model was lower at ~0.86.

Also, we evaluated the pretrained ESM-2 (8 M) model as an additional comparison, restricting optimization to tuning the learning rate of the final MLP classifier rather than fine-tuning the entire model. Therefore, the ESM-2 result is exploratory and is not directly comparable with the fully optimized lightweight architectures. Under this limited setting, ESM-2 (8 M) required substantially longer computation time and achieved an OpenAUC score of around 0.86; it was therefore excluded from subsequent cross-validation. We selected the optimal HP combinations for the four remaining models (Table 1), while the exploratory ESM-2 result is reported separately in Table 2.

**Table 1:** Optimal configurations selected from the HP search.

**Model**	HP	**OpenAUC**	**Time (s)**
RNN	hidden size = 128, layer = 1, lr = 0.0005	0.8646	31.93
GRU	hidden size = 128, layer = 2, lr = 0.0005	0.9119	73.20
LSTM	hidden size = 128, layer = 1, lr = 0.0005	0.9121	89.19
Mamba	d_model = 256, d_state = 8, d_conv = 3, expand = 1, lr = 0.0005	0.8975	62.46

**Table 2:** Exploratory ESM-2 benchmark.

**Model**	HP	**OpenAUC**	**Time (s)**
ESM-2 (8 M)	hidden size = 320, lr = 0.0001	0.8571	17170.0

### Model selection using cross-validation

After identifying optimal HPs, the four most promising models were subjected to a rigorous five-fold cross-validation. As shown in Table 3 and Supplementary Table S8, GRU and Mamba outperformed RNN and LSTM. Although GRU was ~16% slower than Mamba (328.06 s versus 282.49 s), GRU achieved higher OpenAUC (0.9493 versus 0.9469). In addition, the GRU model also demonstrated better versatility by running efficiently on both CPU and GPU environments, whereas Mamba can run only on GPU. Given the strong performance, broader hardware compatibility, and suitability for deployment in resource-constrained settings, the GRU model was selected as the best solution for KO annotation.

**Table 3:** Five-fold cross-validation results for selected deep learning models.

**Model**	**OpenAUC**	**Accuracy**	**AUROC**	**Time (s)**
RNN	0.8867 ± 0.0005	0.5717 ± 0.0185	0.7236 ± 0.0050	140.09 ± 1.81
GRU	0.9493 ± 0.0002	0.9445 ± 0.0004	0.9271 ± 0.0004	328.06 ± 1.01
LSTM	0.9421 ± 0.0009	0.9154 ± 0.0009	0.9085 ± 0.0015	399.25 ± 3.40
Mamba	0.9469 ± 0.0007	0.9451 ± 0.0007	0.9234 ± 0.0008	282.49 ± 1.40

### Threshold calibration for KEGG Orthology classes

To obtain the final prediction model, we first retrained the GRU network using the training split of the final 7:1:2 partition of complete data. When evaluated on the test set in a threshold-independent manner, the model achieved a high OpenAUC of 0.9495, an accuracy of 0.9446 on labeled sequences, and an AUROC of 0.9274 for discriminating between labeled and currently unannotated sequences. For threshold-dependent evaluation, we report F1-score, precision, recall, specificity, and known-sequence coverage. Among these metrics, F1-score, precision, and recall evaluate KO assignment performance on only labeled sequences. Specificity is defined as the proportion of currently unannotated KEGG proteins rejected without receiving a KO assignment, whereas known-sequence coverage is the proportion of labeled sequences receiving a KO assignment.

To convert these probability scores into practical class assignments, we systematically evaluated three distinct thresholding strategies: treating all currently unannotated sequences as a single “operational out-of-scope” class, the OpenMax framework [24], and our proposed joint labeled-unannotated calibration.

We first examined the two common open-set approaches. Treating all currently unannotated sequences as a single class produced the highest specificity (0.9535) and precision (0.8998), but assigned fewer labeled KO sequences, with a recall of 0.8194 and known-sequence coverage of 0.8490. Conversely, OpenMax (*P* = 5) achieved the highest recall (0.8934) and known-sequence coverage (0.9106), but the lowest precision (0.7250) and specificity (0.7567), resulting in an F1-score of 0.8000.

To address these limitations, we implemented joint labeled-unannotated calibration, in which class-specific thresholds were determined from the score distributions of both labeled KO proteins and currently unannotated KEGG proteins. Within this framework, we evaluated thresholds optimized for accuracy, Youden’s index, and F1-score (Table 4). Both accuracy- and F1-optimized strategies performed well. The accuracy-optimized approach achieved a higher F1-score (0.8683 versus 0.8652), precision (0.8962 versus 0.8653), and specificity (0.9319 versus 0.9049) than the F1-optimized strategy. However, the accuracy-optimized method had the drawback of low sensitivity, providing lower recall (0.8420 versus 0.8651) and known-sequence coverage (0.8495 versus 0.8745). Given the importance of recovering enough KO annotations from metagenomic datasets, we therefore retained the F1-optimized thresholds as the default operating point. Because thresholds were optimized independently for each predicted KO class on the calibration data, “F1-optimized” refers to the class-wise optimization objective and does not necessarily produce the highest pooled F1-score on the held-out test set. False-positive and false-negative counts, micro- and macro-F1, and class-size-stratified performance are provided in Supplementary Tables S13 and S14.

**Table 4:** Threshold-calibration performance on the held-out test set.

**Thresholding strategy**	**F1-score**	**Specificity**	**Precision**	**Recall**	**Known-sequence coverage**
F1-optimized	0.8652	0.9049	0.8653	0.8651	0.8745
Accuracy-optimized	0.8683	0.9319	0.8962	0.8420	0.8495
Youden’s index-optimized	0.8460	0.8769	0.8335	0.8589	0.8676
Single unannotated class	0.8578	0.9535	0.8998	0.8194	0.8490
OpenMax (*P* = 5)	0.8000	0.7567	0.7250	0.8934	0.9106
OpenMax (*P* = 10)	0.8193	0.8422	0.7940	0.8463	0.8573

We then evaluated the effect of KO class size by grouping KO classes according to the number of sequence members, defining rare classes containing 1–9 sequences, medium classes containing 10–99 sequences, and abundant classes containing 100 or more sequences. These groups comprised 2390, 6198, and 17 881 KO classes, respectively. Rare KO classes had lower mean F1 than medium and abundant classes in both the full-length mode (42.56%, 73.91%, and 85.08%, respectively) and the fragment mode (47.34%, 54.80%, and 68.39%, respectively). Consistent with this pattern, KO-level analysis showed that F1-scores were more variable among classes with fewer available sequences, while average sequence length showed no obvious relationship with per-KO performance (Supplementary Fig. S2).

The results demonstrate that approaches relying solely on labeled data or aggregating all currently unannotated sequences into a single class yield less balanced decision boundaries. By jointly leveraging labeled and currently unannotated data, our F1-optimized strategy effectively regularizes the thresholds, establishing reliable decision boundaries.

**Figure 4:**
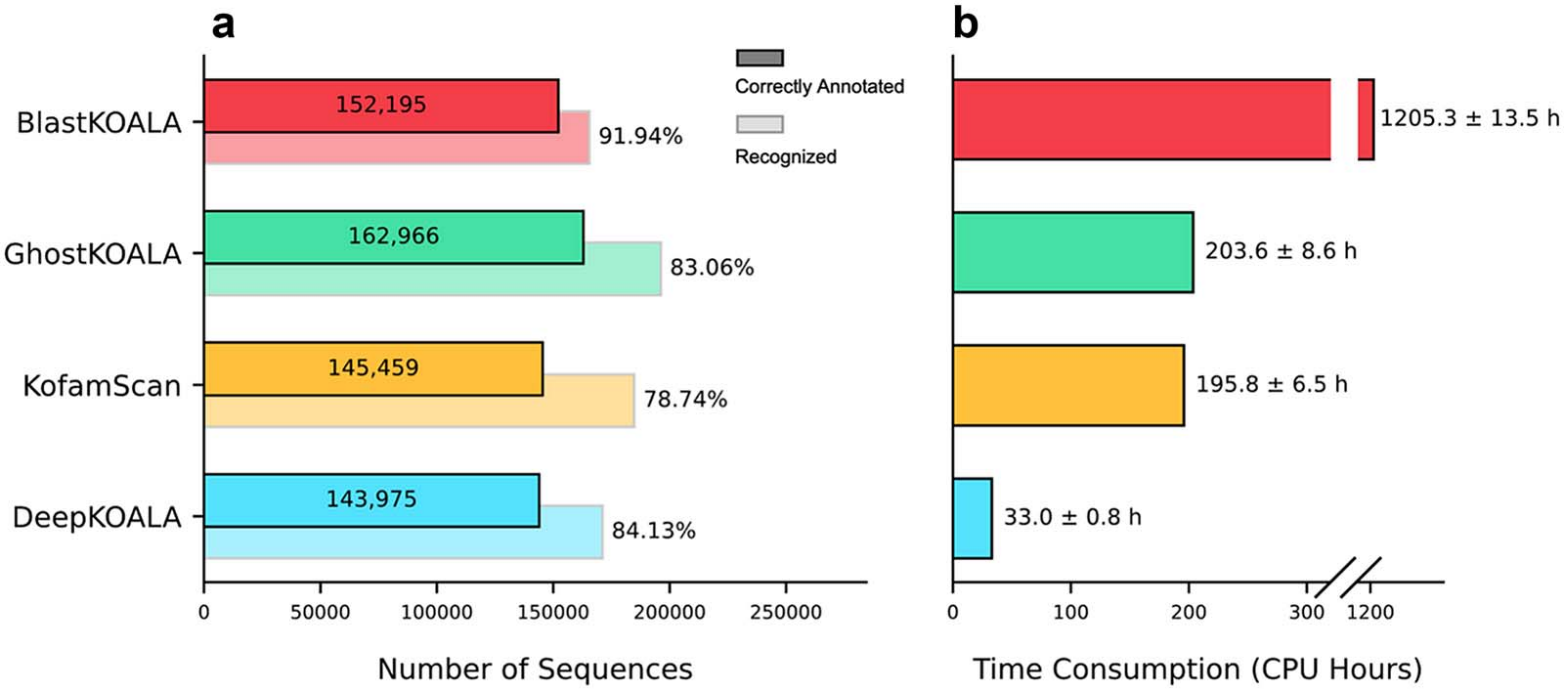
Performance and efficiency comparison with existing tools; (a) comparison of the number of sequences recognized (assigned any KO; light bars) and correctly annotated (dark bars) by DeepKOALA, KofamScan, GhostKOALA, and BlastKOALA on the independent test set of 60 species; (b) mean runtime from three repeated benchmark runs for each tool, highlighting the computational efficiency of DeepKOALA compared to alignment-based methods.

### Benchmarking against homology-based tools

To evaluate the practical performance of our final model, we benchmarked DeepKOALA against three existing annotation tools: BlastKOALA, GhostKOALA, and KofamScan in a CPU environment (see Supplementary Method S3 for hardware, software, and benchmarking details). Because precision alone can favor tools that annotate fewer sequences, we additionally calculated recall, coverage, micro-F1, macro-F1, false positives, false negatives, and currently unannotated rejection rate using a pooled benchmark table that included known-KO records and currently unannotated records for open-set evaluation (Supplementary Table S11), and per-species benchmark metrics are provided in Supplementary Table S12. In this pooled comparison, DeepKOALA achieved precision 84.13%, recall 82.61%, coverage 36.95%, micro-F1 83.37%, and currently unannotated rejection rate 91.27%. BlastKOALA showed the highest precision (91.94%) and micro-F1 (89.58%), whereas GhostKOALA showed the highest recall (93.51%) and coverage (42.37%). DeepKOALA retained higher precision than GhostKOALA (83.06%) and KofamScan (78.74%) (Fig. 4a), and a higher currently unannotated rejection rate than GhostKOALA (89.25%) and KofamScan (89.26%). This high-precision performance is coupled with exceptional computational efficiency. Based on the mean runtime across three repeated CPU benchmark runs, DeepKOALA completed the 60-species benchmark in 32:59:46 and was 36.5 times faster than BlastKOALA, 6.2 times faster than GhostKOALA, and 5.9 times faster than KofamScan (Fig. 4b; Supplementary Table S10). On a single NVIDIA H100 GPU, the same dataset can be annotated in 66 s, providing an additional order-of-magnitude acceleration without altering the prediction outputs. Length-dependent precision profiles on the 60-species test set are shown in Supplementary Fig. S3.

### Application in metagenomic datasets

**Figure 5:**
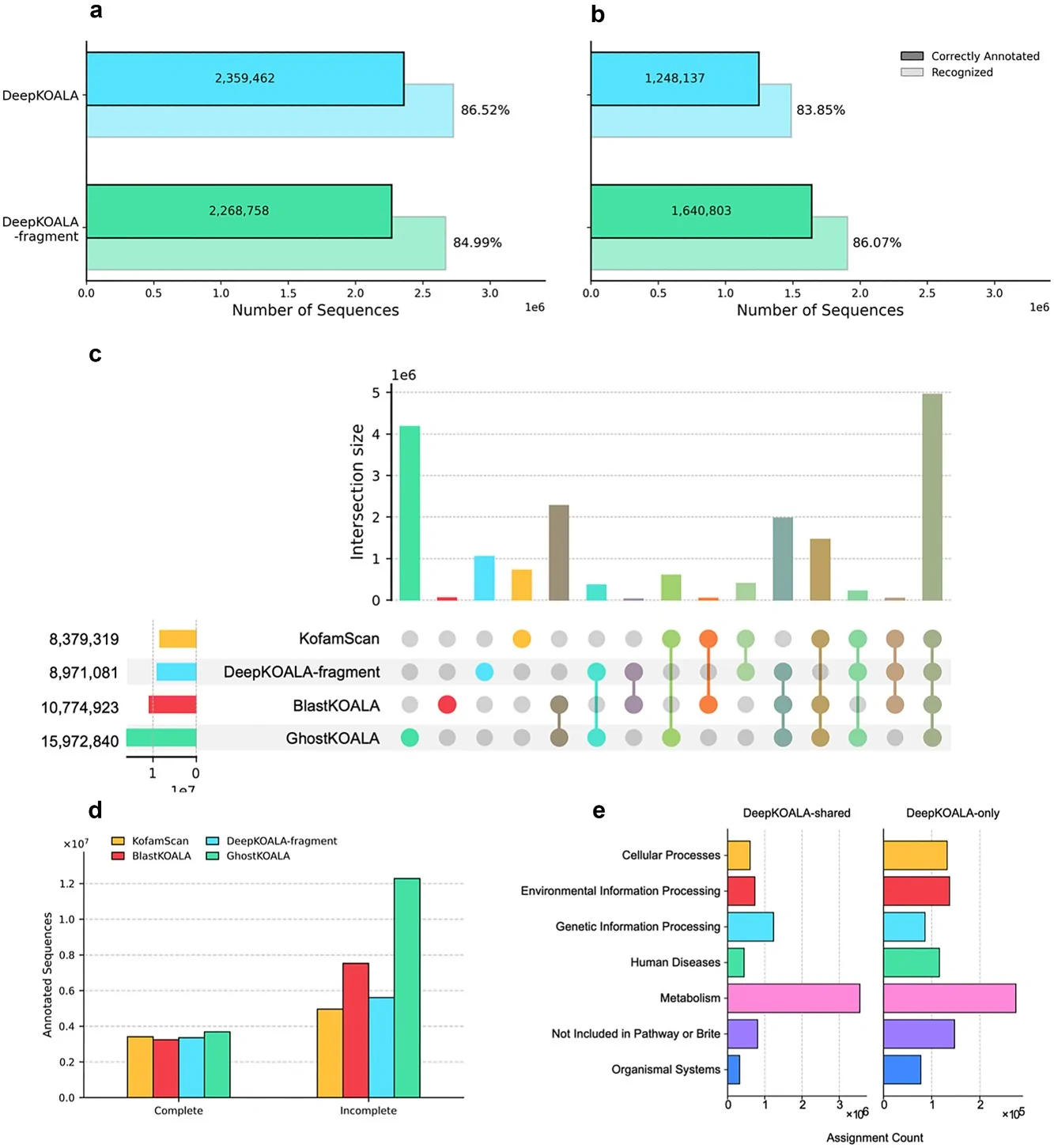
Performance of DeepKOALA on metagenomic datasets; (a) performance comparison between the standard DeepKOALA and the DeepKOALA-fragment model on a full-length protein test set, showing comparable precision; (b) performance comparison on a fragmented protein test set, demonstrating the superior robustness of the fragment model; (c) an UpSet plot showing the intersection of annotations by DeepKOALA-fragment, KofamScan, BlastKOALA, and GhostKOALA on the OM-RGC.v2 dataset; the bars represent intersection sizes, with the single dot column indicating annotations unique to DeepKOALA; (d) numbers of complete and incomplete OM-RGC.v2 proteins assigned a KO by KofamScan, BlastKOALA, DeepKOALA-fragment, and GhostKOALA; the bars represent annotation counts and do not indicate annotation accuracy; (e) KEGG PATHWAY distributions of DeepKOALA-shared and DeepKOALA-only candidate KO annotations; DeepKOALA-shared denotes DeepKOALA annotations also assigned by at least one of KofamScan, BlastKOALA, or GhostKOALA, whereas DeepKOALA-only denotes annotations assigned only by DeepKOALA.

To assess DeepKOALA’s performance on complex, large-scale metagenomic data, we first tackled the issue of sequence fragmentation, a common problem in real metagenomic data caused by low read coverage and errors in sequences. We compared our standard DeepKOALA with a specialized variant model, DeepKOALA-fragment, that was trained on protein sequences cropped to variable lengths. On a test set of full-length proteins, both models showed comparable performance, with the standard DeepKOALA achieving a slightly higher precision (86.52% versus 84.99%) (Fig. 5a). However, when we evaluated models on a fragmentation-simulation test set using randomly cropped sequences, the DeepKOALA-fragment model outperformed the standard model. It not only achieved higher precision (86.07% versus 83.85%) but also recognized substantially more sequences (1 948 803 versus 1 481 137) (Fig. 5b).

Building on this, we applied the DeepKOALA-fragment model to the entire Ocean Microbial Reference Gene Catalog v2 (OM-RGC.v2) [28], which contains 46 828 091 protein sequences. We compared the coverage of DeepKOALA-fragment with KofamScan, BlastKOALA, and GhostKOALA (Fig. 5c). The analysis revealed a substantial core set of 4 950 790 sequences co-annotated by all four tools, indicating substantial agreement among the tools for this subset. Additionally, 1 041 855 sequences were exclusively annotated by DeepKOALA but missed by the other three methods, identifying additional KO annotation candidates for further validation. In terms of runtime, DeepKOALA demonstrated exceptional scalability, completing the task in 38 min and 35 s on a single NVIDIA H100 GPU. For comparison, the same job would require an estimated 1172 CPU hours on a CPU-only system for DeepKOALA, representing a 1850× speedup.

We further evaluated annotation yield according to protein completeness (Fig. 5d). Proteins predicted by Prodigal [29] with both start and stop codons were classified as complete, whereas all others were classified as incomplete. Among complete proteins, KofamScan, BlastKOALA, DeepKOALA-fragment, and GhostKOALA assigned KOs to 3 415 547, 3 246 341, 3 360 489, and 3 688 000 sequences. Among incomplete proteins, the corresponding counts were 4 963 772, 7 528 582, 5 610 592, and 12 284 840.

We further compared the KEGG PATHWAY category distributions of two annotation groups (Fig. 5e). Compared with the DeepKOALA-shared group, the DeepKOALA-only group appeared more broadly distributed across non-metabolic categories, including Cellular Processes, Environmental Information Processing, Human Diseases, and Organismal Systems. To provide an independent supporting assessment of these DeepKOALA-only candidate annotations, we annotated the 1 041 855 DeepKOALA-only sequences with evolutionary genealogy of genes: Non-supervised Orthologous Groups mapper (eggNOG-mapper) v2.1.13 [30–32]. eggNOG-mapper assigned KOs to 247 702 sequences, of which 123 956 had the same KO as DeepKOALA and 123 746 had a different KO. These concordant assignments support a subset of DeepKOALA-only candidate annotations, while the discordant and eggNOG-unannotated sequences indicate that the remaining assignments should be interpreted cautiously.

### Multi-domain mode for multi-domain proteins

Both the standard DeepKOALA model and the fragment model assign a single KO label for each input sequence, which limits their ability to identify multi-domain proteins. To overcome this, we developed a multi-domain mode that combines the GRU model with HMMER in an iterative search strategy, in order to identify multiple domains within a single sequence (Fig. 1d).

We validated this mode using a dataset of 152 RNA polymerase sequences, a family known to include both single- and multi-domain members. As an example, an RNA polymerase (1277 amino acid residues) from *Caldivirga maquilingensis* containing both K03041 (rpoA1; DNA-directed RNA polymerase subunit A') and K03042 (rpoA2; DNA-directed RNA polymerase subunit A") domains was processed using this mode. In this iterative procedure, DeepKOALA first assigned the KO label K03041, and HMMER was then used to delineate the corresponding domain boundaries (residues 15–902). The K03041 segment was subsequently removed computationally before the next iteration. Subsequently, the remaining sequence was re-analyzed and the K03042 domain was correctly identified (residues 910–1275). The results on both single- and multi-domain RNA polymerase sequences support the feasibility of this iterative strategy as a proof of concept (Supplementary Table S9). To evaluate the computational cost, we applied multi-domain mode to the 60-species independent test set. The entire dataset was processed in 76.7 CPU hours, while it was 33 CPU hours without multi-domain mode.

## Discussion

Protein annotation at scale remains a major bottleneck in large metagenomic data analysis, where existing tools struggle with speed, accuracy, or scalability. In this study, we presented DeepKOALA, a deep learning-based tool for KO annotation. By framing KO assignment as an open-set recognition problem, DeepKOALA achieves strong performance on a cross-species benchmark and delivers higher precision than KofamScan (84.13% versus 78.74%) at a similar level of true positive predictions, while it was comparable to GhostKOALA in terms of precision. Although BlastKOALA attained the highest precision (91.94%), DeepKOALA was 36.5 times faster than BlastKOALA on CPU. DeepKOALA annotated the entire OM-RGC.v2 database, containing about 47 million sequences, in ~38 min on a single GPU or 1172 CPU hours on a CPU-only system. Thus, DeepKOALA couples high accuracy with calibrated open-set rejection and high throughput for large genomic and metagenomic datasets.

The advantages of DeepKOALA stem from a fundamental shift in its computational paradigm and careful model architecture selection. DeepKOALA’s inference time is solely dependent on the sequence length and independent of the total number of KO classes, ensuring excellent scalability. Regarding model selection, we considered both large language models (e.g. ESM-2) and lightweight recurrent networks (e.g. GRU). Protein language models can learn rich general-purpose representations from large unlabeled datasets, making them powerful for feature extraction tasks like protein structure prediction. However, for a supervised classification task with tens of thousands of fine-grained labels in our study, the goal is to learn highly specific, discriminative features. In this context, the massive size and quadratic complexity ($O\left({L}^2\right)$) of ESM-2 result in substantial time costs without a corresponding gain in classification performance. Consequently, a lightweight GRU backbone—with linear complexity (*O(L)*) and a small parameter footprint—provides the best accuracy-throughput trade-off for sequence-to-label assignment in our setting.

On a deeper level, KO classification presents challenges that are particularly well-suited to deep learning. The KO system is itself a manually curated, hierarchical classification system designed to represent biological function. This means the degree of internal sequence similarity greatly varies among different KO families, and simple sequence similarity is not always a reliable basis for classification. Deep learning models, on the other hand, can adaptively learn and adjust optimal recognition thresholds based on the underlying distribution of each KO class. This allows them to define the boundaries across KO families beyond simple sequence conservation. To address this variability, we calibrate per-KO thresholds in a semi-supervised manner, which provides an acceptable trade-off between precision, recall, and known-sequence coverage. Compared with a fixed global threshold or OpenMax-style thresholds estimated from labeled data alone, this yields a more stable precision–recall/specificity trade-off. It also aligns with the practice of class-specific adaptive thresholds in KO pipelines.

There are several limitations in the present study. First, as a supervised learning model, its recognition ability is confined to the KO families represented in its training data, and it cannot discover new protein families or remote homologs. Second, performance was lower for rare KO classes than for medium and abundant classes. Third, the fragment model was mainly evaluated using random cropping, which is a controlled simulation and does not fully capture real partial open reading frames (ORFs) with contig-boundary truncation, frameshifts, gene-calling errors, low-quality regions, or short-fragment bias. Fourth, multi-domain mode depends on HMMER and has been validated only as a proof of concept on RNA polymerase sequences, so broader validation across proteins with diverse domain architectures will be needed to establish its general performance. While limitations remain, DeepKOALA demonstrates significant strengths in computational efficiency and achieves a performance comparable to other KO annotation tools. Future work should include temporal validation with later KEGG releases, evaluation on real partial ORFs with different truncation types, broader multi-domain architectures, and error analyses across homologous KO families and poorly represented taxa. DeepKOALA is available on GenomeNet (https://www.genome.jp/tools/deepkoala/) and can be downloaded from our GitHub repository (https://github.com/zhaoxi120/deepkoala).

**Key points**

- DeepKOALA introduces a computationally efficient deep learning framework that formulates Kyoto Encyclopedia of Genes and Genomes Orthology assignment as an open-set recognition task to accurately classify functions while rejecting operational out-of-scope sequences.
- The model achieves annotation precision comparable to standard alignment-based tools but delivers a significant acceleration.
- Validated on a 47-million sequence metagenomic catalog, the framework demonstrates exceptional scalability by completing annotation in ~38 min on a single H100 GPU.
- The software features specialized modes for handling incomplete sequences in metagenomic data and an iterative hybrid strategy for identifying multiple functional domains within a single protein.

## Supplementary Material

supplementary_figure_bbag445

supplementary_table_bbag445

supplementary_table_S3_bbag445

supplementary_table_S4_bbag445

supplementary_table_S7_bbag445

supplementary_table_S8_bbag445

supplementary_table_S9_bbag445

supplementary_table_S11_bbag445

supplementary_table_S12_bbag445

supplementary_table_S13_bbag445

supplementary_table_S14_bbag445

supplementary_method_bbag445

## Data Availability

The DeepKOALA source code, pretrained model weights, inference code, and usage instructions are maintained on GitHub (https://github.com/zhaoxi120/deepkoala) and archived at Zenodo (https://doi.org/10.5281/zenodo.17949963). This archive also hosts the modified KOfam HMM library (excluding proteins from the 654 held-out species; see Supplementary Table S4) and the dataset of 152 RNA polymerase sequences used for multi-domain evaluation. The KEGG GENES FASTA files and KEGG-derived sequence datasets used for model development cannot be redistributed because of KEGG licensing restrictions. The independent benchmark species list and excluded related-species list are provided in Supplementary Tables S3 and S4. The model-development training, validation, and test partitions were random sequence-level partitions of the non-redundant dataset rather than species-level splits. Users can run the released DeepKOALA model on their own legally obtained protein FASTA files.
