# Supplementary material for: DeepKOALA: a scalable deep learning framework for KEGG Orthology assignment
Source: Brief Bioinform. 2026 Aug 19;27(4):bbag445. doi: 10.1093/bib/bbag445 (PMC13489391; doi:10.1093/bib/bbag445)
Supplement: Supplementary file 1 [file supplementary_figure_bbag445.docx]

# Supplementary Figure

**Supplementary Figure S1** – **Sequence Length Distribution of the Full Dataset**

**Supplementary Figure S1 alt-text:** Bar chart showing the sequence length distribution of the full non-redundant dataset across amino-acid length bins.

**Supplementary Figure S2** – **Relationships between KO class size, sequence length, and per-KO F1-score**

**Supplementary Figure S2 alt-text:** Two scatter plots relating per-KO F1-score to KO sequence count and KO average sequence length. The plots show stronger class-size effects than average-length effects.

**Supplementary Figure S3** – **Precision versus sequence length on the 60-species test set**

**Supplementary Figure S3 alt-text:** Line and bar chart showing tool-specific precision across protein sequence-length bins on the 60-species benchmark. BlastKOALA, GhostKOALA, KofamScan, and DeepKOALA are shown as lines, and sequence counts are shown as bars.
